# Mental health needs of tribal college students in Araku Valley

**DOI:** 10.1007/s44192-024-00105-1

**Published:** 2024-10-28

**Authors:** Rishitha Swathi Karuturi, Suneetha Kandi

**Affiliations:** Department of Applied Psychology, GITAM University, Visakhapatnam, India

**Keywords:** Mental health needs, Tribal students, Aboriginals, Adolescents, Young adults

## Abstract

The indigenous tribal population in India, often residing in remote and underserved regions, represents a marginalized minority with limited access to healthcare, particularly mental health services. Despite the pressing need, there is a scarcity of research focusing on the mental health challenges faced by adolescents and young adults within these communities. This study addresses this gap by examining the mental health needs of tribal college students in the Araku Valley region of Andhra Pradesh, India. The study sample comprised 291 participants (118 males, 173 females), representing adolescents and young adults from various tribal backgrounds. Utilizing the Students Counseling Needs Questionnaire and the General Health Questionnaire-30, the study identified alarmingly high prevalence rates of mental health concerns, including uncontrollable disturbing thoughts (73.18%), loneliness (62.19%), depression (57.04%), phobias (54.97%), and suicidal ideation or attempts (39.16%). Notably, the study revealed a trend of decreasing mental health needs with advancing age, suggesting that adolescents are more vulnerable than young adults. Furthermore, significant gender differences were observed, with female students reporting higher family-related needs compared to male students. These findings provide critical insights into the mental health challenges faced by tribal college students and have important implications for policy development and educational practices aimed at addressing the unique needs of this population. Educators at tribal institutions can use these findings to address the mental health needs of tribal students.

## Introduction

Mental health is a state of well-being that enables people to cope with the stresses of life, to realize their abilities, to learn well and work well and to contribute to their communities. Mental health is an integral component of health and well-being and is more than the absence of mental disorders [1]. Despite advances in the medical field, we still struggle to accept mental illness in our societies, and in many communities, it remains a stigmatized ‘taboo’ concept. According to a global report by the Institute for Health Metrics and Evaluation in 2019, 12% of the world’s population is reported to be experiencing mental disorders [2]. The National Mental Health Survey of India 2015–16 revealed that the mental morbidity of individuals above the age of 18 years is 10.6% [3]. The situation has worsened, as 25% more of the population has reported experiencing anxiety and depression since the COVID-19 pandemic [4]. The COVID-19 pandemic highlighted the significant shortcomings of global healthcare systems, particularly in the realm of mental health care, revealing weaknesses that were previously overlooked. In India, the crisis led to a surge in mental health issues, revealing inadequacies in mental health services and response systems [5]. There is a global average of 13 mental health professionals for every 100,000 people, according to the Mental Health Atlas 2020 report given by the World Health Organization in 2021 [6]*.* An average of 1.93 mental health professionals are reported for every 100,000 people in India [7]. As a lower middle-income country [8], India spends approximately 4 rupees per person on mental health expenditures [7]. In 1982, India implemented national-level programs such as the National Mental Health Program and the District Mental Health Program to address the mental health burden. Despite the government’s efforts, progress seems painfully slow to have a significant on-ground impact.

The need for mental health services continues to increase, whereas the availability of and access to mental health care remain scarce. The government, along with other organizations, is taking initiatives to address this challenge. The National Mental Health Policy (NMHP), launched in 2014, provides guidelines for decreasing the treatment gap and disease burden. Rashtriya Kishor Swasthya Karyakram (RKSK)—2014 is a national program focused on the holistic development of adolescents in India. The Mental Healthcare Act (MHA), launched in 2017, helps formulate laws and ensures that they are implemented across states. It focuses on the rights of people with mental illnesses but does not involve any specialized approach to adolescents’ mental health. The Ministry of Health and Family Welfare, Government of India, launched the National Suicide Prevention Strategy in 2022 to address the rising rates of suicide, particularly among individuals aged 15–29 years. This initiative was driven by the significant increase in anxiety, depression, and suicide rates in India following the COVID-19 pandemic in 2019 [9]. It is a step in the right direction that helps alleviate the mental health burden, as it is known that psychological problems in adolescence persist into adulthood [10].

Studies have shown a substantial mental health burden in the Indian population, with adolescents and young adults being particularly vulnerable, highlighting the critical need for targeted mental health interventions. According to Gautham and colleagues, 10.6% of the participants, aged older than 18 years, reported having mental health morbidity, and approximately 150 million Indians needed mental health intervention. Among them, adolescents and young adults are the two specific groups that are often in high need of mental healthcare services [11]. They are likely the most affected by mental health challenges, contributing significantly to disability-adjusted life years among different age groups [12]. Adolescents and young adults, particularly those aged 16–22 years, are experiencing substantial lifestyle shifts that are often accompanied by significant mental health challenges. These changes, stemming from the transition from a protective school environment to a more independent and demanding college setting, are marked by new responsibilities, evolving social dynamics, and increased pressure, all of which can contribute to psychological distress and the development of mental health issues [13, 14].

A recent Pan India study revealed that 18.8% of college students in India had considered suicide at some point in their lives, with 12.4% having done so in the past year and 6.7% having attempted it. Additionally, 33.6% of the participants reported moderate to severe depression, whereas 23.2% experienced moderate to severe anxiety [15]. In another study of 1038 late adolescent college students of Andhra Pradesh, aged 17–19 years, 45.6% reported loneliness, 57.4% experienced uncontrollable disturbing thoughts, 50.4% struggled with depression, 31.9% had nightmares, and 17.6% reported suicidal thoughts/attempts [16].

Recent studies consistently highlight that adolescent girls face more significant mental health challenges than their male counterparts do. For example, Van Droogenbroeck and colleagues reported that girls experience higher rates of depression and anxiety, particularly as they transition through adolescence [17]. This trend is further supported by Cela-Bertran and colleagues, who reported that emotional distress is more prevalent among girls, as measured by the Gender Adherence Index (GAI) [18]. Additionally, a large-scale cross-national study by Campbell, Bann, and Patalay [19], which included over 566,000 adolescents across 73 countries, confirmed that girls consistently reported more mental health issues, such as depression and anxiety, than boys did.

The findings from studies on nontribal college students reveal alarmingly high rates of mental health challenges, such as suicidal ideation, depression, and anxiety. Given these statistics, investigating the mental health scenario within tribal populations, who face additional socioeconomic marginalization and limited access to mental health care, is crucial. Exploring the mental health needs of these tribal communities is essential for understanding the broader mental health crisis in India. India has a total population of 121.1 crores, and the scheduled tribes of India comprise 10.4 crores of that population [20]. Article 342 of the Indian constitution provides the specifications that make up a tribe or a tribal community. For a community to be identified as a scheduled tribe, it must adhere to being a tribe with primitive traits, distinctive culture, shyness of contact with society at large, geographical isolation, and backwardness. The scheduled tribes constitute 8.6% of the country’s population and remain marginalized. Among the 8.6% tribal population in India, 11.3% live in rural areas, whereas only 2.8% are reported to live in urban areas [20].

The distinctive nature of tribal communities that sets them apart can be a double-edged sword. On the one hand, these characteristics foster strong community bonds and a rich cultural heritage. On the other hand, they can lead to isolation from mainstream society, making it challenging to access education, healthcare, and economic opportunities. This isolation can also hinder the effective implementation of development initiatives, resulting in slow progress despite government efforts. Although the government has initiated policies and plans to develop tribal areas, progress has been painfully slow. The literacy rate of scheduled tribes in India is 58.96%, whereas the country’s literacy rate is 72.99%. A total of 17.7% of the literacy gap is observed among individuals in scheduled tribes and the total population of India [20]. Andhra Pradesh is one of the states with literacy rates of scheduled tribes that are less than the country’s average literacy rate for the Scheduled Tribe population, which is only 49.2% [20]. According to Manna and colleagues  [21], poverty in tribal regions can be linked to inequalities. A few other studies have identified the economic conditions of tribes, along with the geographical locations of tribal villages, as a roadblock to educational attainment [22]. Child marriages, tribe location, inadequate education facilities, and lack of family support are other social factors contributing to low literacy rates [23].

Among research studies on the mental health of tribal populations in India, Ali and Iqbal assessed the mental health status of tribal adolescent boys in Jharkhand via the Strengths and Difficulties Questionnaire [24]. Their study reported that 5.1% of the students had emotional issues, 9.61% had conduct problems, 4.23% exhibited hyperactivity, and 1.41% experienced significant peer problems. In a study on tribal people of Madhya Pradesh attending a local health camp over 18 years of age, 7.3% were diagnosed with common mental illnesses, whereas 32.7% were diagnosed with severe mental illnesses. The majority of the patients in the study had anxiety disorders, whereas 24.8% had psychotic spectrum disorders [25]. In the Idu Mishmi population, an isolated tribal group from Arunachal Pradesh, the suicide attempt rate is 14.22%, with females being at greater risk. This study, which used the Patient Health Questionnaire on adolescents and family members of people who committed suicide, reported depression at 8.26%, anxiety syndrome (6.42%), alcohol abuse (36.24%), and eating disorders such as binge eating (6.42%) and bulimia nervosa (1.38%) [26].

A meta-analysis by Rajkumar and colleagues revealed that the pooled prevalence of depression among rural adolescents in India was 27%, whereas anxiety disorders had a pooled prevalence of 26%, and suicide prevalence was 9% [27]. This study revealed that gender was significantly associated with only social anxiety disorders, without a significant gender association with other mental health problems. A cross-sectional survey conducted in the Indian states of Gujarat, Tamil Nadu, and Meghalaya among 623 indigenous tribal adolescents (11–19 years) revealed that the prevalence of psychiatric morbidity in these adolescents is nearly twice the national average [28]. The study found that 6.4% of the adolescents experienced mood disorders, 9.1% had neurotic and stress-related disorders, 6.3% suffered from phobic anxiety disorder, 2.7% had alcohol use disorder (AUD), 2.7% experienced behavioral and emotional disorders, and 2.2% were diagnosed with obsessive‒compulsive disorder. This study also revealed that psychiatric morbidity was greater among older girls aged 15–19 years, with a prevalence rate of 66.6%.

Most of the literature points to the need for further research into the mental health needs of the tribal population. A recent systematic review revealed that research on mental health issues in tribal populations in India is sparse, with existing studies primarily focusing on a narrow range of concerns like alcoholism, anxiety, depression, and suicide [29]. This suggests that the broader spectrum of mental health issues in these communities is underexplored and potentially underestimated. To address these gaps, the current study aimed to assess the prevailing mental health needs of tribal students. Mental health concepts can be adapted to the tribal context by considering the communal and holistic approaches inherent in these populations. According to the World Health Organization, mental health is a state of mental well-being that enables individuals to manage life's stresses, realize their potential, and contribute meaningfully to society [30]. It encompasses a spectrum of experiences, from mental wellness to mental health conditions that involve significant distress or impairment. Thus, this study aimed to assess the mental health needs of tribal college-going adolescents and young adults, focusing on various dimensions, including academic challenges, personality and adjustment issues, social interactions, family dynamics, and clinical concerns. By examining these distinct areas, this research seeks to provide a comprehensive understanding of this population’s specific mental health needs. A measure to assess general mental health was also used to identify psychological distress in tribal college students to gain insight into their overall mental well-being. This study also aimed to explore sex differences and examine the relationships among age, mental health needs, and psychological distress among tribal students.

## Methods

### Study population

A quantitative methodology was employed to collect and analyze the data for the current study. The sample population for this study was selected from the Araku Valley region of the Alluri Sitaram Raju district, Andhra Pradesh, India. Andhra Pradesh (AP) accounts for 5.68% of the country’s tribal population [20]. The commonly found scheduled tribes in AP are Valmiki, Dhulia, Paiko, Gadaba, Kotia, Savara, Konda Dhora, Kuttiya, and Dongira. The state also has several Particularly Vulnerable Tribal Groups (PVTGs), such as Chenchu, Bodo Gadaba, Gutob Gadaba, Dongira Khond, Kultia Khond, Kolam, Konda Reddi, Khond Porja, and Kondasavara. PVTGs are the most marginalized sections of India's tribal population, identified by the Ministry of Tribal Affairs, characterized by preagricultural technology, low literacy, and stagnant or declining population growth. Owing to the presence of many tribes in AP, it was the first state that the government declared Scheduled Areas in 1950 [31]. However, no studies on the mental health needs of tribes in this region have been reported. The state of Andhra Pradesh was selected for this study because of its significant and diverse tribal population, particularly in regions such as the Araku Valley, which is home to a variety of indigenous groups.

### Screening of participants

After receiving approval from the Ethics Committee, the researcher approached the relevant educational authorities in Araku Valley, shared the details and procedures of the study, and secured permission to proceed with the research. Purposive sampling was employed to select the study participants. The participants were selected on the basis of the following inclusion criteria: belonged to a tribal community, lived in the tribal region, attended junior/senior colleges based in the tribal region, were able to communicate in English, and voluntarily agreed to participate in the study.

The participants who met the inclusion criteria were then screened for alcohol and drug usage prior to inclusion in the study. Alcohol consumption in the indigenous population is known to be high compared with that in the general population. A total of 49.9% of tribal men and 14.1% of tribal women are reported to consume alcohol in India [20]. As substance abuse leads to various interpersonal, emotional, and financial issues, it is possible that individuals consuming alcohol or drugs might have a high prevalence when reporting mental health needs. For this reason, the students were initially screened using the *Tobacco, alcohol, prescription medication, and other substances (TAPS) tool.* The TAPS tool was used to identify participants for the use of alcohol, tobacco, prescription drugs, and other illicit substances in the past year. The questionnaire has been found to be valid and accurate at detecting substance use disorders [32]. A total of 304 participants were given the TAPS tool, and 4.28% of the participants (13 students) tested positive for substance use. The researcher excluded the participants who tested positive and continued the study, resulting in a total of 291 participants. According to the sample size calculations, a sample of 273 participants was deemed sufficient to provide a reliable estimate of the population within ± 5% of the true value with a 90% confidence level.

### Ethics approval and consent to participate

The study was presented to the GITAM Institutional Research Ethics Committee, where the research objectives, methodology, and ethical considerations were thoroughly reviewed. Upon careful evaluation, the committee granted formal approval, ensuring that the study adhered to ethical guidelines and institutional standards. Following this approval, all students willing to participate in the study were provided with informed consent forms. They were briefed on the study's purpose and methodology, assured of their confidentiality, and informed that their participation posed no risk. Participants were also made aware of their right to withdraw from the study at any time. To further protect their privacy, all participant information was anonymized and securely stored in encrypted digital formats, accessible only to authorized personnel. Informed consent was obtained with clear communication about how their data would be used, protected, and kept confidential. Ethical guidelines, including those related to confidentiality, were strictly followed throughout the research, ensuring the privacy and confidentiality of the tribal population involved in the study.

### Assessment tools and administration

All participants who passed the screening test were included in the study. The assessment tools used were in English, as the students were from educational institutions where English is the medium of instruction. The subjects were administered the tools as a group in one of their classrooms, wherein one of the researchers was available throughout the assessment to clarify doubts, if any. Each participant first completed a demographic sheet, and the researcher administered the Student Counseling Needs Questionnaire (SCNQ) to the participants. Subsequently, the participants were given the General Health Questionnaire-30 (GHQ-30) to complete. After the response protocols were collected, the researcher ensured that the data were securely placed in sealed envelopes to maintain data integrity.

*Goldberg's 30-item General Health Questionnaire (GHQ-30)* measures minor psychiatric disorders with the physical elements removed. It assesses an individual’s current state, ability to function, and appearance of new symptoms. The GHQ is suitable for use in both adolescents and young adults worldwide. The scale consists of 30 items, and the modern Likert scoring method was used for scoring. The GHQ-30 was used to help detect psychological distress and provide insights into the overall mental well-being of tribal students.

The *Student Counseling Needs* (SCNQ) questionnaire assesses five dimensions of mental health needs among students for whom they seek counseling. It assesses academic needs related to performance pressures and study challenges, personality/adjustment needs concerning personal growth and adaptation to college life, social needs involving issues with social interactions and relationships, family needs focusing on the impact of family dynamics on students’ well-being, and clinical needs aimed at identifying symptoms of mental health conditions that may require professional intervention [16]. The questionnaire has a reliability of 0.86, and it has been used in Indian adolescent and young adult populations [33]. This questionnaire has 52 items and is measured using a 3-point scale: 1-not needed, 2-somewhat needed, and 3-most needed. The score was 0 for ‘not needed’, 1 for ‘somewhat needed’, and 2 for ‘most needed.’

### Statistical analysis

The IBM Statistical Package for Social Sciences (SPSS) 22 was used for statistical analysis. Before the analysis was conducted, data cleaning was performed, including addressing missing data through appropriate imputation methods. Descriptive statistics, such as the median, mean, and standard deviation, were calculated. Prevalence rates of mental health needs among tribal subjects were computed. Pearson correlation coefficients were computed to assess the relationships between age and dimensions of mental health needs and also between age and GHQ-30 scores. Additionally, independent sample t tests were conducted to explore sex differences in the variables included in the study.

## Results

### Demographics

The study's final sample included 291 participants (118 boys and 173 girls) belonging to two junior colleges and two senior colleges. Among them were 178 adolescent junior college students and 113 young adult senior college students. The demographic details of the participants, consisting of 291 tribal students, are presented in Table 1. Most participants were 17 years of age, accounting for 37.5% (n = 109) of the sample, followed by 18-year-olds at 25.1% (n = 73). The gender distribution revealed that 59.5% (n = 173) were female, and 40.5% (n = 118) were male. With respect to education, 61.2% (n = 178) were in junior college, whereas 38.8% (n = 113) were in senior college. Most participants (63.6%, n = 185) reported a family income of 0–50,000 Indian rupees. The sample included students from various tribes, with the largest group being Khodu (22.3%, n = 65), followed by Porja (16.2%, n = 47) and Gadaba (12.4%, n = 36). The other tribes represented smaller sample proportions, such as Konda Reddi (11.7%, n = 34), Valmiki (11.7%, n = 34), and others.

**Table 1:** Demographic details of the participants

		Frequency^1^	Percentage
Age (years)	16	6	2.1
	17	109	37.5
	18	73	25.1
	19	19	6.5
	20	12	4.1
	21	31	10.7
	22	17	5.8
	23	18	6.2
	24	2	0.7
	25	4	1.4
Sex	Male	118	40.5
	Female	173	59.5
Education	Junior College	178	61.2
	Senior College	113	38.8
Family Income (Indian Rupees)	0–50,000	185	63.6
	50,000–1,00,000	89	30.6
	1,00,000–5,00,000	17	5.8
Tribe	Khodu	65	22.3
	Konda Reddi	34	11.7
	Savara	27	9.3
	Kuttiya	19	6.5
	Gadaba	36	12.4
	Desaya	15	5.2
	Porja	47	16.2
	Goudu	14	4.8
	Valmiki	34	11.7

1 = Total number of participants (N) = 291

### Prevalence of mental health needs

With respect to the mental health needs for which the students sought counseling, all the top five needs were found to be academically related (see Table 2). The students’ responses indicated that strengthening memory-improving techniques was the top need for which they sought counseling, with an 80.06% prevalence rate. A total of 24.39% of the students agreed that it was the ‘most needed’ need, and 55.67% agreed that it was ‘somewhat needed’. Setting relevant goals is another need, with the second-highest prevalence rate of 78.34%. Improving writing skills, effective learning methods, and coping with work pressure had prevalence rates of 76.62%, 73.53%, and 73.53%, respectively. Coping with distractions while studying and managing exam stress were also a few needs that most students reported, with 71.12% and 69.06%, respectively. A total of 62.19% of the students also indicated a need for career counseling.

**Table 2:** Prevalence of Counseling Needs in the tribal college students

Needs	% Most Needed	% Somewhat needed	% Total
Academic needs			
Memory Improvement Techniques	24.39	55.67	80.06
Set Relevant Goals	27.83	50.51	78.34
Improve Writing Skills	25.42	51.2	76.62
Effective Learning Methods	25.08	48.45	73.53
Coping with Work Pressure	17.52	56.01	73.53
Coping with Distractions while Studying	25.08	46.04	71.12
Managing Exam Stress	20.96	48.1	69.06
Problems due to Medium of Instruction	18.55	49.48	68.03
Understanding Subject Matter	20.61	45.7	66.31
Attention/Concentration Improvement	18.9	45.36	64.26
Frame Proper Time Table	16.83	47.42	64.26
Efficient Study Habits	21.64	41.92	63.56
Career Guidance	26.8	35.39	62.19
Increase Work Commitment	24.39	35.73	60.12
Increase Self-Motivation in Studies	22.68	35.39	58.07
Note Taking Skills	18.9	37.45	56.35
Interest in Studies	30.24	23.71	53.95
Personality/adjustment needs			
Enhance Decision Making Skills	26.8	42.61	69.41
Overcome Lethargy	20.96	45.7	66.66
Overcome Fear of Failure	21.3	38.14	59.44
Anger Management	15.8	43.29	59.09
Improve Self Discipline	18.21	39.17	57.38
Increase Self Control/Patience	21.3	34.36	55.66
Increase Self Confidence	21.99	30.92	52.91
Gain Meaning in Life	17.86	28.86	46.72
Social needs			
Conflict Management–Resolving Problems	16.15	55.32	71.47
Overcome Infatuations	15.12	56.01	71.13
Overcome Shyness	18.55	49.82	68.37
Enhance Communication Skills	25.42	42.26	67.68
Assertiveness–Ability to say ‘NO’	21.64	44.67	66.31
Overcome Loneliness	15.8	46.39	62.19
Coping with Broken Relationships/Death	17.86	39.51	57.37
Overcome Peer (Friends) Pressure	14.43	41.92	56.35
Respect–Self & Others	18.21	28.86	47.07
Compassion–Ability to think about others	14.77	31.27	46.04
Family needs			
Financial Problems	17.52	54.63	72.15
Coping with Family Disputes	20.96	50.51	71.47
Overcome Homesickness	16.83	45.36	62.19
Adjustment with other relatives	17.52	44.32	61.84
Lack of Family Support	17.18	42.61	59.79
Adjustment with Siblings	16.15	38.83	54.98
Adjustment with Parents	14.77	39.86	54.63
Clinical needs			
Uncontrollable Disturbing Thoughts	15.8	57.38	73.18
Tiredness/Weakness	12.71	57.04	69.75
Sleeplessness	9.96	58.76	68.72
Nightmares	15.46	45.36	60.82
Health Problems	15.8	42.95	58.75
Coping with Depression	13.4	43.64	57.04
Phobias	14.08	40.89	54.97
Sleepy	11.34	39.51	50.85
Suicidal Attempts/Suicidal Thoughts	9.96	29.2	39.16
Bad Habits (Drugs/Alcohol/Smoking)	8.93	27.14	36.07

Enhancing decision-making skills and overcoming lethargy were the top personality/adjustment needs for which the students sought counseling (69.41% and 66.66%, respectively). Regarding the top social needs, 71.47% of the students indicated that they needed help in conflict management. Overcoming infatuations is another need, with a high prevalence rate of 71.13%. Given the socioeconomic levels of tribal communities, it is not surprising that 72.15% of students indicated a need for counseling in terms of financial problems. A total of 71.47% of the students reported difficulty coping with family disputes. Overcoming homesickness, adjusting with other relatives, and lacking family support were the top family needs, with prevalence rates of 62.19%, 61.84%, and 59.79%, respectively.

Uncontrollable disturbing thoughts are reported by 73.18% of the participants as the top clinical need, with 15.8% of the students agreeing that it is ‘most needed’ counseling need and 57.38% of the students agree that they are the ‘somewhat needed.’ Tiredness/weakness, sleeplessness, and nightmares are also rated high among clinical needs, with prevalence rates of 69.75%, 68.72%, and 60.82%, respectively. 62.19% of the students reported loneliness and 57.04% of the students reported difficulty coping with depression. A total of 13.4% of the students reported it to be the ‘most needed’ mental health need, whereas 43.64% of the students rated it as ‘somewhat needed.’ Suicidal attempts/Suicidal thoughts had a prevalence rate of 39.16%, with 9.96% of the students rating it as ‘most needed’ and 29.2% of the students rating it as ‘somewhat needed.’ Another mental health need largely reported from the clinical dimension was phobias, with a prevalence of 54.97%. A total of 14.08% of the students regarded it as ‘most needed’, whereas 40.89% of the students regarded it as ‘somewhat needed.’

### Relationship of age with other variables

Another objective of the study was to determine the relationships between the age of the participants and their SCNQ and GHQ-30 scores. The mean age of the students was 18.73 years, with a standard deviation of 2.12. The GHQ-30, which measures general health, had a mean score of 8.92 and a standard deviation of 7.19. With respect to student counseling needs, the academic dimension had the highest mean score of 15.25 (SD = 7.65), followed by the social dimension, with a mean of 7.92 (SD = 3.74). The personality/adjustment dimension had a mean score of 6.31 (SD = 3.81), the clinical dimension had a mean score of 6.96 (SD = 3.40), and the family dimension had a mean score of 5.58 (SD = 2.97). Table 3 shows the relationships between age, GHQ-30 scores, and various dimensions of counseling needs among tribal students using Pearson correlation. The analysis revealed a significant negative correlation between age and personality needs (r = − 0.231, *p* < 0.001), social needs (r = − 0.174, *p* = 0.003), family needs (r = − 0.269, *p* < 0.001), and total counseling needs (r = − 0.204, *p* < 0.001). The GHQ-30 score, which measures general health, shows a weak positive correlation with total counseling needs (r = 0.037, *p* = 0.528), although this correlation is not statistically significant. Other relationships, such as those between age and academic needs and between age and clinical needs, were not significant.

**Table 3:** Relationships between Age, GHQ-30 score, and Needs Dimensions of Tribal Students

Variable		Academic Needs	Personality Needs	Social Needs	Family Needs	Clinical Needs	Total Counseling Needs	GHQ
Age	Pearson Correlation	− 0.098	− 0.231	− 0.174	− 0.269	− 0.107	− 0.204	0.037
	Sig. (2 tailed)	0.094	0.000***	0.003**	0.000***	0.069	0.000***	0.528

**p < 0.01. ***p < *0.001*

### Gender differences

This study also intended to explore gender differences, if any, in tribal college students. Table 4 shows the mean differences between male and female tribal students in terms of various dimensions of mental health needs. The results indicate that male students had a mean score of 15.10 for academic needs, which was slightly lower than that of females, who had a mean score of 15.35; however, this difference was not statistically significant (*p* = 0.779). For personality/adjustment needs, males scored 6.40, whereas females scored 6.25, which was not a significant difference. Similarly, with respect to social needs, males had a mean score of 7.84, and females had a mean of 7.97 (*p* = 0.783). The clinical needs dimension for males, with a mean of 6.77, and females, with a slightly greater mean of 7.10, was also not significant. However, with respect to family needs, females reported a higher mean score of 5.88 than males did at 5.16, with a statistically significant difference (*p* = 0.047).

**Table 4:** Means and SDs of male & female tribal students on mental health needs dimension with *t* values

Dimensions	Sex^1^	Mean	Std. Deviation	*t* value	Sig. (2-tailed)
Academic	Male	15.10	7.68		
	Female	15.35	7.65	− 0.280	0.779
Personality/Adjustment	Male	6.40	4.08		
	Female	6.25	3.63	0.334	0.739
Social	Male	7.84	4.07		
	Female	7.97	3.51	− 0.276	0.783
Family	Male	5.16	2.91		
	Female	5.88	2.99	− 1.997	0.047*
Clinical	Male	6.77	3.55		
	Female	7.10	3.29	− 0.819	0.413

1 = Male (118) and Female (173). * = *p* < 0.05

## Discussion

This study addresses a significant gap in the understanding of mental health needs among tribal college students in India, a group often overlooked in research. While existing studies have largely focused on a limited range of issues, such as alcoholism, anxiety, depression, and suicide, this research broadens the scope by examining a wider spectrum of mental health concerns within this population. It adopts a comprehensive approach, assessing various dimensions, including academic challenges, personality and adjustment issues, social interactions, family dynamics, and clinical concerns. This study aligns with the World Health Organization's holistic definition of mental health, which encompasses not only the absence of mental illness but also the ability to manage life's stresses, realize one's potential, and contribute meaningfully to society [30].

The findings of this study reveal high prevalence rates of academic-related needs among tribal students, paralleling results from a previous study by Kandi [16] conducted on the general college student population. This trend among tribal college students may stem from their enrollment in the same state government curriculum in Andhra Pradesh, suggesting that academic pressures are consistent across different student demographics. The current study also assessed mental health needs, such as coping with depression, loneliness, and suicidal thoughts, which have been documented in earlier research. For example, depression had a prevalence rate of 57.04% in the current study, which aligns with findings from Kandi [16] (50.4%) and Cherian [15] (33.6%). Similarly, Singh [26] reported that 8.26% of adolescents reported depression, and Rajkumar’s [27] meta-analysis reported a 27% prevalence among Indian rural adolescents.

Suicidal thoughts and attempts were reported by 17.6% of the students in the Kandi [16] study and 18.8% in the Cherian [15] study, with Singh and colleagues [26] reporting a 14.22% suicide attempt rate among the Idu Mishmi tribal population. In contrast, Rajkumar et al. [27] reported a lower 9% prevalence rate among rural adolescents. Additionally, 54.97% of the students in the current study sought counseling for phobias, a significantly higher percentage than the 6.3% reported in a study by Gharat et al. [28] on phobic anxiety disorder. The current study also revealed that 73.18% of the students reported uncontrollable disturbing thoughts and that 56.35% had difficulty overcoming peer pressure, whereas Ali and Iqbal [24] reported much lower rates, with only 1.41% of tribal adolescent boys experiencing significant peer problems and 5.1% facing emotional issues. With respect to substance abuse, 36.07% of the students in the current study identified bad habits (drugs/alcohol/smoking) as mental health needs for which they sought counselling. Comparatively, studies by Singh [26] and Gharat [28] found similar rates of alcohol abuse among the tribal adolescent population, with 36.24% and 2.7% lower prevalence rates, respectively.

Overall, this study revealed high prevalence rates of mental health needs across various dimensions, indicating significant psychological distress among adolescents and young adults. These findings support previous research by Gautham and colleagues [11], Mokdad [12], Cleary [13], and Chadda [14], who explored the mental health challenges faced by these age groups. Addressing the mental health needs of tribes is part of the holistic healthcare that the government aims to provide. It is highly recommended that the government and other organizations working toward tribal welfare look into the mental health of adolescents and young adults, as the psychological stress they carry now can persist into adulthood. Any psychological interventions designed to address tribal populations, especially tribal youth, must be rooted in indigenous tribal culture and specifically designed to meet their mental health needs, as reported.

This study revealed that among tribal college students, the mental health needs decreases with age, especially concerning personality/adjustment issues, social interactions, and family dynamics. Compared with their older peers, adolescent tribal students presented more significant mental health needs. This trend may be due to the challenges associated with adolescence, such as identity formation, social pressures, and adjusting to new responsibilities, which are more pronounced during the transition period into college life. Many researchers have noted that college-age students have higher rates of mental health issues [11, 12], especially when they transition from school to college [13, 14]. As students age and progress through their college years, they might develop better coping mechanisms, gain more independence, and become more accustomed to their environment, leading to a reduction in mental health needs.

With respect to gender differences, consistent with the findings of some prior studies conducted with nontribal students [17–19] and those with tribal students [27, 28], the current study revealed that women tribal college students have higher prevalence rates for mental health needs than men do. A study by Gharat et al. on indigenous tribal adolescents found older adolescent girls reported more psychiatric morbidity than their counterparts did [28]. The current study also revealed that women and men differ significantly with respect to family-related needs with women reporting higher, whereas there is no such difference with respect to needs in other dimensions. Rajkumar et al. [27] reported significant differences in only one disorder in their study of rural adolescents, while there were no gender differences with respect to other mental health problems. The observed higher prevalence of mental health needs among female tribal college students than among their male counterparts may be attributed to cultural norms, socioeconomic disadvantages, and additional domestic responsibilities that disproportionately affect women in tribal communities.

This study contributes significantly to understanding the mental health needs of the indigenous tribal population in the Araku Valley of Visakhapatnam, AP, India. Research on the mental health of tribal college students is lacking. By focusing on a population that is often underrepresented in mental health research, this study highlights critical issues specific to tribal communities. The findings can equip educators at tribal institutions with valuable insights to effectively address the unique mental health needs of their students. Another strength, lies in its comprehensive assessment of mental health needs across various dimensions, aligning with the World Health Organization's holistic definition of mental health, which includes managing life's stresses, realizing potential, and contributing meaningfully to society. However, a notable limitation is the lack of control for confounding variables, expect for substance use, which could have provided a more nuanced understanding of mental health needs within this unique cultural and environmental context. While the primary objective was to gain an initial understanding of prevalent mental health issues, future studies should consider controlling other confounding variables to increase the accuracy of the findings. Additionally, the study’s reliance on quantitative methodology, while valuable for identifying broad trends, might have overlooked deeper, qualitative insights that could reveal more about the mental health crisis among tribal youth. Therefore, future research should adopt a mixed methods approach to capture the breadth and depth of these issues. Moreover, the study's focus on a specific geographical location—Araku Valley—limits the generalizability of its findings. Expanding future research to include tribal populations from diverse geographical regions would provide a more global perspective on the mental health needs of tribes, especially among adolescents and young adults, and help tailor interventions more effectively to different tribal communities.

## Conclusion

This study provides critical insights into the mental health needs of tribal college students in the Araku Valley, Andhra Pradesh, India, highlighting the specific challenges faced by this often over looked population. The findings reveal high prevalence of mental health issues, particularly in areas such as academic stress, depression, loneliness, and suicidal thoughts, with notable gender differences and a decrease in mental health needs as students age. These results not only align with those of several previous studies but also emphasize the importance of culturally sensitive mental health interventions tailored to the unique needs of tribal communities.

## Data Availability

The data supporting this study’s research findings will be provided by the corresponding author upon reasonable request.
